# Dynamic intrusion detection for internet of drones using glowworm swarm optimization

**DOI:** 10.1038/s41598-026-44789-7

**Published:** 2026-03-20

**Authors:** Radha Kowtharapu, C. H. Surya Kiran, P. Aruna Kumari

**Affiliations:** 1 Department of CSE, JNTU, Kakinada, Andhra Pradesh India; 2 Department of CSE, Koneru Lakshmaiah Education Foundation, Green Fields, Vaddeswaram, Guntur Dist, Andhra Pradesh India; 3 Department of CSE, JNTU-GV College of Engineering, Vizianagaram, India

**Keywords:** Dynamic feature selection, Glowworm swarm optimization, Intrusion detection system, Internet of drones, Real-time threat detection, Resource-constrained environments, Engineering, Mathematics and computing

## Abstract

The Internet of Drones (IoD) is spreading at excessive speeds that have posed a challenge in operation in that should be both secure and viable in terms of identifying the threats of intrusion and avoiding them in a dynamic and resource limited setting. In the given paper, a novel technique called DIGS-RTD is presented and this is a fresh framework using the bio-inspired Glowworm Swarm Optimization (GSO) algorithm to dynamically pick features. The luminescent communication based GSO approach can be applied to multimodal optimization problems, with its approach to the optimization of multimodal components appropriately applicable to locate a variety of intrusion patterns, as well as very complex intrusion patterns. The proposed framework has an active feature selection mechanism that aims to reduce the size of the feature subset that is utilized in intrusion detection to minimize the number of computational load without compromising the accuracy of detection.An efficient classification model has been integrated with the selected features to detect the intrusions in real-time. The system is compared with state-of-the-art bio-inspired approaches, namely Sea Turtle Foraging Algorithm (STFA), against a wide range of performance metrics, including detection accuracy, false positives, false negatives, processing time and memory usage. The scalability and adaptability of the framework in handling the increasing diversity of IoD with the data flow rate and network size are also evaluated. Through experimental evaluation, the approach GSOSTFA proves of having superior trade-off between computational cost and detection performance by decreasing false positives and processing time against baseline methods. The dynamic nature of the IoD and the framework’s ability to adapt to changing environments highlights the potential of this as a powerful method of securing IoD operations against the constantly evolving landscape of cyber threats. This represents an important advance in the field of IoD security, with efficient and scalable real-time capabilities for intrusion detection in next-generation drone networks.

## Introduction

Internet of Drones (IoD), to improve the synchronization of UAVs (unmanned aerial vehicles) used for logistics, disaster management, surveillance, and other possible implementations However, the increased reliance on IoD systems has resulted in several significant cybersecurity challenges because these networks are especially vulnerable to various intrusion attacks^1^. For secure functioning in IoD environment, IDS is played an essential role against these threats. These recent undertakings have not only highlighted the necessity of creating mechanisms that can efficiently and cost-effectively manage the dynamic attributes of IoD networks, but also that can still function in scenarios of resource scarcity^2^.

By lowering the dimensionality of data, subordinating the computational costs, and ultimately increasing the accuracy of intrusion detection feature selection is an essential procedure in constructing efficient intrusion detection systems. To this end, many techniques such as genetic algorithms, PSO, and bio-inspired techniques are used for the optimization and feature selection^3^. While these methods have achieved a level of cross-domain formalization, they are also widely divergent in their stability and performance when generalized with possible dynamic and multimodal nature of the IoD settings. The Glowworm Swarm Optimization (GSO) Algorithm is a very recent bio-inspired optimization algorithm which offers a new possibility for solving these problems, because of its luminescence-based interaction mechanism GSO is able to discover heterogeneous intrusion patterns^4,5^.

Another new feature extraction technique is the Sea Turtle Foraging Algorithm (STFA), which is considered as the state-of-the-art approach that increase the intrusion detection performance of IoD IDS^6^. However, STFA in large-scale, resource constrained IoD environments still has limited scalability and computational efficiency in terms of metrics^7^. Studies if done on the effectiveness of bio-inspired algorithms gives a reference point that can help in justifying the use of such new upcoming optimization techniques like GSO that will bring out much better performance in the terms of detection accuracy or resource optimization. Because IoD intrusion detection has diverse properties as well as needs, GSO is a suitable optimization algorithm because it is flexible to dynamic optimization problems^8^.

This paper proposes DIGS-RTD (Dynamic intrusion detection in the Internet of Drones using Glowworm swarm Optimization for RTD (Real Time Threat Detection)), a new framework to secure IoD networks. To provide competitive detection accuracy with wide applicability within resource-limited environments, the proposed framework introduces the use of the GSO algorithm to implement dynamic feature selection to reduce the computational cost of the network^9^. Compared to the Sea Turtle Foraging Algorithm (STFA) and additional bio-inspired approaches, the system is tested and achieves material advancements in detection accuracy, false positive rate, false negative rate, processing time, and memory usage. We target IoD security with this study which is a robust and scalable solution that provides intrusion detection of the next-generation networks, a narrowing research area^10^.

Despite tremendous advances in intrusion detection systems (IDS) of the IoD networks, most of the currently used strategies do not respond to the demands of the dynamic but resource limited environment. The traditional IDS technologies are usually anchored on fixed feature selection schemes, which are not in line with dynamic characteristics of the intrusion in IoD networks.Bio-inspired optimization algorithms, including Particle Swarm Optimization (PSO) and Genetic Algorithms (GA), have shown promise in feature selection; however, these techniques do not efficiently optimize multimodal problems, as those frequently arise in IoD system operations. Several works of literature on the Sea Turtle Foraging Algorithm (STFA) have delivered progress in contrast; unfortunately, the STFA’s scalability and computational efficiency remain limited in the context of large-scale IoD applications. Filling these gaps is crucial to develop dynamic feature selecting approaches to reduce computing burden while enabling high-quality intrusion detection to protect the security of an efficient IoD operation.

This research is motivated by the integration of IoD networks upon modern applications such as logistics, surveillance, and disaster management, and the evolving and growing threats towards cybersecurity on such systems. Introducing security measures such as an Intrusion Detection System (IDS) into such networks is challenging, due to the dynamic and decentralized nature of IoD networks, requiring solutions that are capable to adapt and that consume as few resources as possible. Overall, inspired by the numerous bio-inspired algorithms with great potential, the Glowworm Swarm Optimization (GSO) algorithm, which possesses a luminescent communication mechanism and several unified features that can be used to implement multimodal optimization, seems to be an attractive design choice for a feature selection algorithm to be used in the field of intrusion detection system (IDS). The research presented in this paper intends to complement GSO capabilities in such a way that a strong and scalable intrusion detection framework can be designed for IoD environments. This may help improve the real-time threat detection mechanisms without costing too much in terms of computation, which is important to successfully achieve secure IoD networks for mission-critical applications.

GSO and STFA are both existing metaheuristics, but this paper adds by reworking and hybridizing them to the changing demands of selecting features in IoD settings, and proposing a multi-objective model of fitness and showing a steady rise in detection accuracy, false alarms and computational efficiency. Therefore, the input is all systems-level and application-driven as opposed to algorithmic reinvention. The key contribution of this work is presented as follows:

- Proposed a new real-time intrusion detection system to Internet of Drones (IoD) setting to accommodate dynamically changing network dynamics and resource limitations.
- Created a hybrid bio-inspired optimization method that Glowworm Swarm Optimization (GSO) and Sea Turtle Foraging Algorithm (STFA) that took advantage of dynamically choosing compact and relevant feature subsets.
- Formulated a fitness definition that simultaneously optimises the accuracy of intrusion detection and the complexity of a feature subset to minimise the computational cost without compromising detection performance.
- Incorporated a lightweight supervised classifier to use the optimized feature subsets to guarantee low-latency and memory consumption rate that fits low-latency real-time IoD applications.
- Performed general assessments on CICIDS2017 dataset in terms of various performance measures (ROC-AUC, FPR, recall, and processing time) and proved to be more accurate, better with scaling, and more robust than the current methods.

## Literature review

The newly introduced Internet of Drones (IoD) has complicated the issue of cybersecurity with its constantly developing environment that is subject to resource limitations. Intrusion detection systems (IDSs) are, therefore, important as far as securing internet of things (IoD) networks is concerned. Hadi et al. (2024)^11^ proposed UAV-CIDS, in which a collaborative IDS was presented with Feedforward Convolutional Neural Networks (FFCNN) and InfoGain to select features and counter all zero-day attacks with encouraging outcomes and reduced time elapsed during real-time conditions. Yet, their approach had certain limits in the nature of UAVs that they themselves employed since it could not be easily scaled to larger IoD networks. In the same manner Ihekoronye et al. (DroneGuard)^12^, in 2024 following the tackling of GPS spoofing and DoS attacks, focused on decision tree and SHAP interpretability data to determine the explainable IDS framework. Although its lightweight design, but the very limited integration with deep learning models restrained its ability to use with complex intrusion scenarios​.

Bio-inspired optimization algorithms have demonstrated considerable capability of improving how we mitigate the complexity and overhead of applying intrusion detection in the IoD environment. The applicability of GSO optimization is further explained by Patel and Singh (2020)^13^, where they concluded that GSO is useful for multimodal optimization, which is most suited for dynamic intrusion patterns in IoD networks. Additionally, Lee et al. (2022)^14^, which investigated use of Sea Turtle Foraging Algorithm (STFA) for feature selection in IoT security domains, highlighting its usefulness within resource-constrained situations. Nonetheless, due to the scalability problem of STFA in large-scale IoD applications, adaptive and efficient optimization strategies are needed.

Some of the latest work on IDS performance enhancement in IoD is hybrid optimization based. Zhao et al. (2024)^15^, presented a scalable IoD intrusion detection framework combining bio-inspired algorithms for managing big data sets while achieving a high detection rate. Their work extends to the IoD real-time needs as well pointing toward the necessity of integrating real-time IDS mechanisms to address changing threats (Wang and Zhang, 2023)^16^. These studies highlight the need for balancing computational efficiency with strong detection performance, which continues to be a major research challenge.

Furthermore, various deep learning models have been proven to have a great potential in IDS. Ashraf et al. (2019)^17^, proposed a hybrid B-LSTM method of intrusion detection in the IoD setting, which yielded similar results but failed in practice because of the lack of standardized data. On the same note, Elsharif et al. (2024)^18^ reviewed machine learning methods in UAV security and emphasized the disadvantage of a high rate of false positives in the anomaly-based detection schemes. The key understanding of this kind of information is that paradigms to trade detectability effectiveness with operational costs and be scaleable and flexible in developing IoD scenarios are required.

Table 1 discusses the similar problem statement comparison of existing approaches that were worked in different ways.

**Table 1:** comparison of the existing approaches addressed the similar problem statement differently.

Authors	Contribution	Methodology Used	Dataset Used	Applications	Limitations
Hassan Jalil Hadi et al. (RTCID)^19^	Proposed UAV-CIDS, a collaborative IDS for UAV networks using FFCNN to enhance detection of zero-day attacks.	Feedforward Convolutional Neural Network (FFCNN), feature selection via InfoGain.	UAVIDS (real-world UAV network traffic).	Cybersecurity for UAV networks, real-time incident response handling.	Limited to specific UAV types; lacks extensive testing on diverse datasets.
Vivian UkamakaIhekoronye et al. (DG)^20^	Developed DroneGuard, a lightweight ML and XAI-based framework for intrusion detection in drone networks.	Decision Tree, SHAP for interpretability, SMOTE for class balancing, RSCV for hyperparameter tuning.	Real-time GPS dataset and cybersecurity dataset.	Detection of GPS spoofing and DoS attacks in drones.	Limited focus on ML models; lacks deep learning integration for complex scenarios.
Syeda Nazia Ashraf et al. (SMF)^21^	Introduced a modular IoT-enabled drone framework integrating ML/DL for robust cybersecurity measures.	Hybrid B-LSTM and LSTM techniques.	CICIDS2017, KDDCup 99 datasets.	Smart drone security, intrusion detection in IoT-enabled drones.	Lack of standardized data collection hierarchy; gaps in real-time deployment.
Raghad A. AL-Syouf et al. (MLA)^22^	comprehensive review of ML-based IDS for UAVs, analyzing threats, methodologies, and datasets.	Reviewed supervised and unsupervised ML models (e.g., SVM, CNN, LSTM).	Multiple datasets including NSL-KDD, CICIDS2017.	Intrusion detection and UAV cybersecurity strategies.	High false alarm rates in anomaly-based detection methods; challenges in dataset diversity and quality.

Intrusion detection of mobile and dynamic networks has become one of the important topics of study in recent years. Mobile ad hoc networks (MANETs) have also been studied to have light and secure detection schemes to improve resource limitations and security issues. Banking on blockchain-enhanced structures, e.g., have been suggested to optimize integrity and security of intrusion detection systems in MANETs^23^. Ensemble learning has been also used in network security where it has been shown that when multiple classifiers, such as Random Forest, AdaBoost and Gradient Boosting have been pooled together, they can indeed offer considerable improvement in detection rates in dynamic settings^24^. The strong ability to detect sophisticated intrusion patterns in a wide variety of network topologies has been achieved through such approaches, as well as reliability in MANET routing, such as straddling path recovery-based strategies being deployed to support consistent communication in cases of node mobility and node failure^25^. These solutions assure that the reliability of the network is maintained even in inappropriate circumstances.

Power-ware routing protocols have as well been introduced to maximize MANET resource consumption. On-demand multipath has been applied to multipath routing to even out the energy usage and network performance^26^. On the same note, a multi-path routing would have been optimized using hybrid methods in dynamic and cluster-based MANET, which strives to optimize the throughput by which the delay is minimized^27^.Lastly, the trust-based and energy-related models have been incorporated in secure MANET-IoT environments to be sure that the choice made in the network takes into account both the trustworthiness and resource limits of the network^28^. These models can be applied especially to the case of IoT-enabled drone networks, where dynamic topology and the limited energy resources are posing special problems.These studies collectively highlight the importance of combining security, reliability, and optimization strategies for intrusion detection in dynamic networks, motivating the development of GSOSTFA, which leverages Glowworm Swarm Optimization to perform real-time threat detection in Internet of Drones (IoD) networks.

Recent studies show that edge intelligence, secure distributed learning, and aerospace systems are rapidly convergent. High-mobility wireless reliability is improved by sophisticated modulation and detection networks^29^, while autonomous navigation robustness is increased by pulsar-based time-delay estimation^30^. Trust in cooperative robotic swarms is bolstered by privacy-preserving localization frameworks^31^, and real-time aero-engine diagnostics show that edge AI is feasible for safety-critical platforms^32^. For robust mobile ecosystems, hierarchical malware detection is combined with deep neural operator optimization to speed up adaptive onboard inference^33,34^. Cooperative autonomy in airspace with many obstacles is advanced by multi-UAV trajectory planning^35^, and scalable cross-layer orchestration is made possible by mission-driven satellite–terrestrial scheduling^36^. Reusable aircraft are supported by intelligent reentry planning^37^, while effective edge inference architectures lower latency and energy overhead^38^. Intelligent retrieval in embedded systems is improved by LLM-augmented multimodal ranking^39^.

Concurrent developments in communications and sensing enhance aerial intelligence: sub-terahertz UAV channel modeling enhances air–ground links^40^, infrared weak-target detection increases surveillance sensitivity^41^, and causal visual navigation enhances perception^42^. While adaptive UAV control manages uncertainty^43^, machine learning-based taxiing perception facilitates smart airports^44^. Autonomous sensing is expanded through energy-aware IoT data collection^45^ and aerial semantic segmentation^46^. Satellite connectivity services^6^ and SAR-based aircraft detection^47^ improve worldwide coverage. Durability and dependability are increased through federated aerial learning^48^, proactive cyber threat detection^49^, deep-space relay optimization^50^, stratospheric airship control^51^, and life-cycle prediction^52^. The following technologies demonstrate a trend toward intelligent, resilient, and scalable aerospace–cyber ecosystems: quantum-inspired air traffic optimization^53^, hypersonic adaptive control^54^, radar-assisted beamforming^55^, federated scheduling^56^, swarm fault tolerance^57^, perturbation-resilient recognition^58,59^, synchronized event control^60^, ransomware analytics^61^, hybrid monitoring^62^, VTOL safety control^63^, and autonomous microservice diagnosis^64^.

## Experimental setup

The experiments were performed on a high-performance machine configuration that consisted of an Intel i7 processor with 16GB RAM and 1 TB storage that allowed the experiments to execute smoothly and provide feedback on the analyses. For the training and test stages, we used a standard dataset for intrusion detection, called the CICIDS2017 dataset. The dataset includes both normal and malicious traffic (DoS – Denial-of-Service) and can be used specifically in DoS evaluation process. The data was split into training and testing sets with 70:30 proportions, while ensuring that both normal and attack traffic were well-represented in both stages. Visual studio IDE was harnessed to maximise the C + + 23.0 built in capabilities to streamline coding process and debugging process. Method: OPNET has been used for modeling the IoD environment and assessing the impact of DoS attacks on drone’s communication. Glowworm Swarm Optimization (GSO) was incorporated into the detection framework to dynamically identify and neutralize the threats in realtime. For this evaluation, accuracy, precision, recall, and F1 are the metrics in order to verify the system performance against the ground truth of the dataset. The used dataset is^23^.

**Fig. 1:**
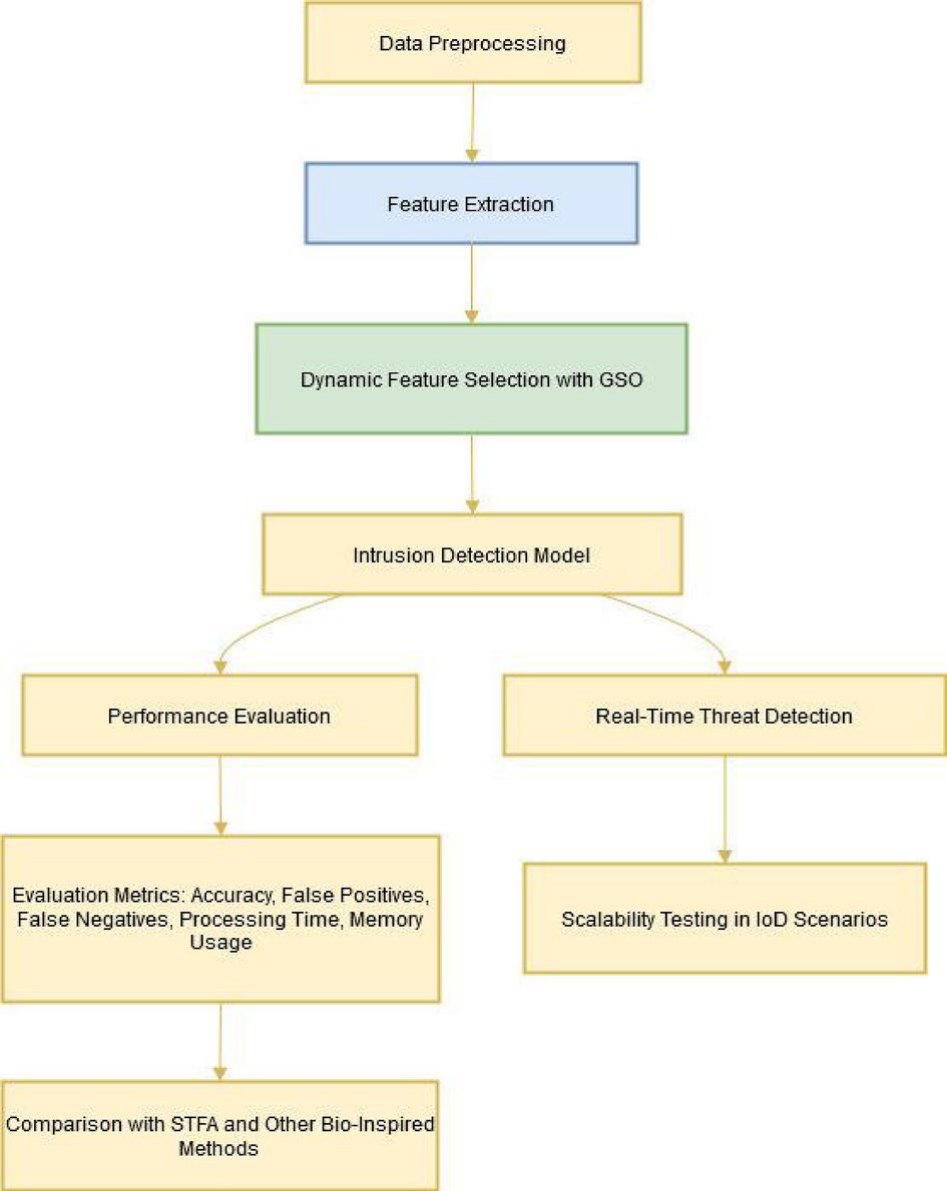
Architecture of DIGS-RTD Framework for Dynamic Feature Selection and Intrusion Detection in IoD Environments.

Figure 1 shows the Model architecture of DIGS-RTD framework for real time feature selection and attacks detection over Internet of Drones (IoD). It involves processing of un-canonical data i.e. cleaning & normalizing raw IoD (instrument of disclosure) data to render it ready for analysis. Then comes Feature Extraction, which is where relevant features are identified and extracted from the data, which makes this process more efficient and representational. In the framework, GSO based Dynamic Feature Selection (GSO-DFS) is taken as a running element, as it applies the Glowworm Swarm Optimization methodology to dynamically select the most important features, thus enhancing the computation efficiency in terms of increasing the verification accuracy. The resulting features are passed into the Intrusion Detection Model that classifies the corresponding data identifying its own threat. The model has two main outputs: Real Time Threat Detection, which is the responsiveness to effectively recognize an intrusion during the attempted infiltration, and Performance Evaluation, the metrics used to assess this performance are accuracy, false positive and negative rates, processing time and memory required. Scalability Testing in IoD Scenarios ensure that the flexibility of the system is available, which also achieves optimally regardless of the width and thinness of the network and also the large-scale data flow. The comparative outcome to other methods is the existence of Sea Turtle Foraging Algorithm (STFA) as well as other bio-inspired approaches, where the superiority of the offered framework is demonstrated. It is through this structure that strong and efficient mechanism of intrusion detection is provided in the dynamic and adaptive working of Heterogeneous Internet of Devices.

The CICIDS2017 data belongs to the range of the largest benchmarks to test intrusion detection systems (IDS) on the controlled environment, and it is also hosted by the Canadian Institute for Cybersecurity (CIC). It consists of network traffic observations of a realistic network simulation model, which was designed to emulate the activity of the real corporate networks of the present day.

**Fig. 2:**
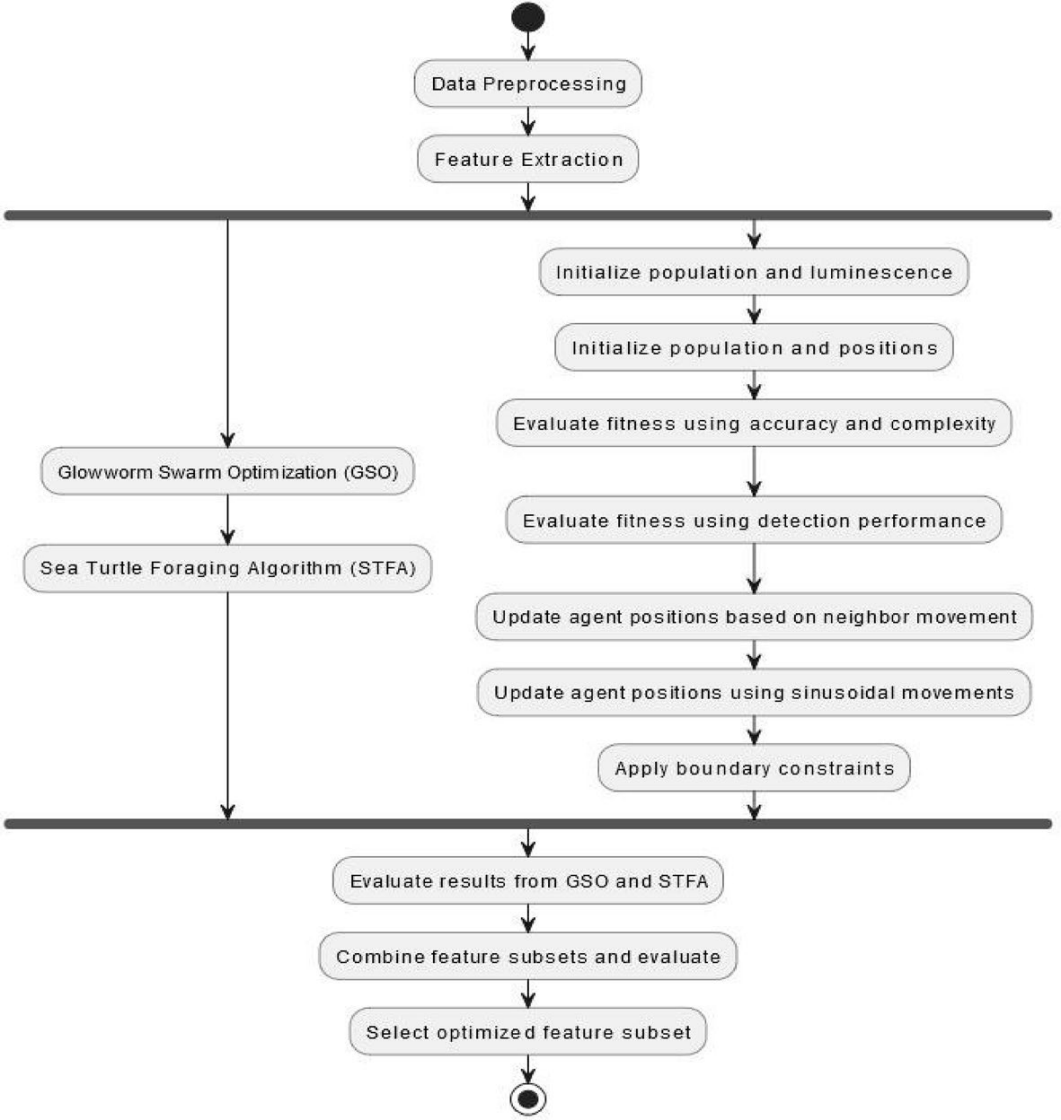
Activity diagram of the proposed approach.

The data will be complete with a normal traffic and malicious data: the brutality attack, denial of service (DoS), intrusion, botnet and web attack. CICIDS2017 contains rich and labelled data for machine learning and cybersecurity research, with more than 80 features obtained from network flow data such as timestamps, protocols, and byte counts. The defined environment is close to reality, and the included up-to-date variety of attack types make it an excellent input to develop and verify intrusion detection algorithms on complex, real-world network environments. The workflow diagram show in Fig. 2 is for selection of dynamic features and detection of intruders based on Glowworm Swarm Optimization (GSO) and Sea Turtle Foraging Algorithm (STFA) is shown in an activity diagram. Experience in Data Preprocessing: Raw Data Cleaning & Preparation: The raw data is prepared for feature extraction in order to identify the relevant attributes for intrusion detection. The workflow then splits into two parallel processes. Initialization of the population and luminescence value is done in the GSO process, where accuracy and computational complexity are checked according to the fitness using the updated agent position through neighbour interactions. Meanwhile, based on detection performance the fitness is evaluated based on the positions and population initialized during the STFA process, agents update their position with sinusoidal movement until fitness convergence occurs where boundary constraints are applied as to maintain the positivity of agent positions. The conclusions are then merged after both GSO and STFA process independently followed by combining feature subsets and assessing based on respective performance metrics, including accuracy and efficiency. At last, the selected feature subset, which is optimized, is chosen as the output and the process is terminated. Therefore, the proposed process provides systematic procedures for valuable and precise construction of features used for anomaly detection in IoD scenarios.

## Proposed algorithm

The hybrid algorithm is the combination of GSO and STFA, and is used to solve the optimization of features in a dataset D= {${x}_{1}$, ${x}_{2}$,……… ${x}_{n}$ } with a feature space F={ ${f}_{1},{f}_{2}$, …….${f}_{m}\}.$To begin with, there is an initial set of agents (each correspondent to a candidate feature subset) placed on the map randomly. GSO is used to fitness estimation and its own given custom function which gauges detection accuracy and feature complexity in that way that a luminescence update is applied to lead the swarm movement. This assists in guiding agents towards optimal solutions, while continuously narrowing its search range using a decaying exploration factor β\betaβ. At the same time, the STFA portion also refines the search through adjusting new weighted agent positions based on the sine-based perturbations and the fitness gradients to exploit valuable regions in the solution space. Position and speed limits are put in place to make sure that valid characteristics are selected. At both iterations, the two terms can be aggregaged to evaluate and revise the population by relying on the fact that the best feature subset F∗F^*F∗ is identified with regard to the top-scoring detector. This is a hybrid method that involves some exploitation and exploration to make more relevant and less redundant the features and acquire a small but effective set of features to make a classification choice or detection.

**Proposed Algorithm: GSO-STFA: Glowworm Swarm Optimization and Sea Turtle Foraging Algorithm** 
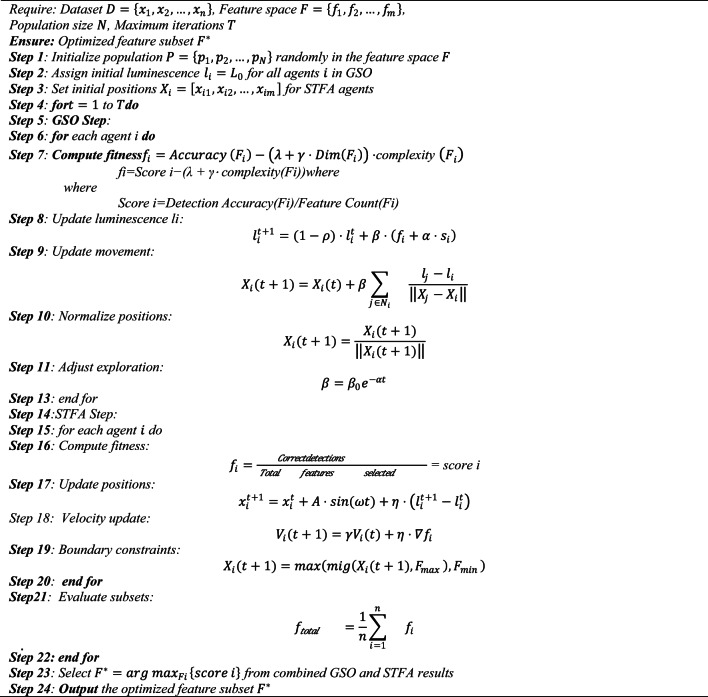


### Mathematical model of the proposed framework

In the proposed framework of DIGs-RTD, intrusion detection in Internet of Drones (IoD) is formulated as an optimization and classification problem of features, in which the goal is to achieve maximum detection behavior and minimum computation cost. The framework combines both Glowworm Swarm Optimization (GSO) and Sea Turtle Foraging Algorithm (STFA) to create a dynamic optimal choice of features, among the high-dimensional network traffic data.

Let the intrusion detecting data set be denoted as:

$$D=\left\{\right({x}_{i},{y}_{i})\mid i=\mathrm{1,2},\dots,N\}$$

where ${x}_{i}\in{R}^{m}$denotes the feature vector of the ${i}^{th}$sample with $m$total features, and ${y}_{i}\in\left\{\mathrm{0,1}\right\}$represents the class label (normal or attack). The complete feature space is defined as:

$$F=\{{f}_{1},{f}_{2},\dots,{f}_{m}\}$$

Each optimization agent corresponds to a candidate feature subset ${F}_{i}\subseteq F$. The goal is to identify an optimal subset ${F}^{*}$such that:

$${F}^{*}=arg{max}_{{F}_{i}\subseteq F}\hspace{0.25em}J\left({F}_{i}\right)$$

where $J\left({F}_{i}\right)$is the fitness function.

#### Fitness function

The fitness combines accuracy of detection with compact size of feature subsets together and is defined as:

$$J\left({F}_{i}\right)=\frac{Acc\left({F}_{i}\right)}{\mid{F}_{i}\mid}-(\lambda+\gamma\cdot\mid{F}_{i}\mid)$$

where $Acc\left({F}_{i}\right)$ is the classification accuracy obtained using feature subset ${F}_{i}$, $\mid{F}_{i}\mid$ denotes the number of selected features, $\lambda$ and $\gamma$ are regularization coefficients that penalize large feature subsets.

This is to promote a large validity of detection in the minimization of redundancy and computation cost.

#### Glowworm swarm optimization (GSO) modeling

Each glowworm agent $i$ is associated with a luminescence value ${l}_{i}\left(t\right)$, which is updated at iteration $t$as:

$${l}_{i}(t+1)=(1-\rho)\cdot{l}_{i}\left(t\right)+\alpha\cdot J\left({F}_{i}\right)$$

Wher e$\rho$ is the luminescence decay constant,$\alpha$ is the enhancement factor.

The movement of the agents into direction of neighbouring higher luminescence agents in a dynamic decision radius r i (t). The update of movements is determined as:

$${x}_{i}(t+1)={x}_{i}\left(t\right)+s\cdot\frac{{x}_{j}\left(t\right)-{x}_{i}\left(t\right)}{\parallel{x}_{j}\left(t\right)-{x}_{i}\left(t\right)\parallel}$$

where $s$ is the step size and ${x}_{j}$ is the position of a brighter neighboring agent.

#### Sea Turtle Foraging Algorithm (STFA) modeling

To enhance exploitation, STFA refines agent positions using sinusoidal perturbation:

$${x}_{i}(t+1)={x}_{i}\left(t\right)+{v}_{i}\left(t\right){v}_{i}(t+1)=\omega\cdot{v}_{i}\left(t\right)+\eta\cdot sin(\pi\cdot r)$$

Where ${v}_{i}\left(t\right)$ is the velocity of the agent, $\omega$ is the inertia factor, $\eta$ controls the foraging intensity, $r\in\left[\mathrm{0,1}\right]$ is a random number.

Boundary constraints are applied to ensure valid feature selection:

$${x}_{i}(t+1)\in[\mathrm{0,1}{]}^{m}$$

#### Optimal feature subset selection

At each iteration, feature subsets obtained from both GSO and STFA are evaluated using the fitness function. The globally best solution is selected as:

$${F}^{*}=arg{max}_{i}J\left({F}_{i}\right)$$

The resulting optimal feature set ${F}^{*}$ is then presented to the intrusion detection classifier to detect threats in real-time with a reasonable level of accuracy, low false positive rates, and a lower computational cost, which makes the proposed scheme applicable to dynamic and resource-restricted IoD settings.

## Results and discussions

### Dataset description

The suggested DIGS-RTD framework was experimentally evaluated using CICIDS2017 which is a well-known benchmark dataset published by the Canadian Institute of Cybersecurity (CIC). The dataset is a set of 748,016 labeled network traffic instances which have over 80 flow-based characteristics, including, but not limited to, packet statistics, protocols, time-based, and measurements on the bytes. These characteristics qualify the dataset to be used to evaluate the intrusion detection systems in dynamic and heterogeneous settings such as the Internet of Drones (IoD). The final GSO–STFA frameworks produced more efficient feature subsets which were classified with a Decision Tree classifier because it was suitable to use in the real-time IoD environment.

In order to have a credible and non-biased performance assessment, the dataset was separated into 70% of training data and 30% testing data. In this split, a stratified sampling method was used to maintain the original class distribution in that both normal and attack traffic samples were represented in the training and testing sets proportionately. This strategy was beneficial in ensuring that the dataset was balanced without any artificial oversampling or undersampling methods and therefore artificial bias was minimized and the natural features of network traffic in the real-world were retained.

The CICIDS2017 dataset was chosen because it requires a variety of attacks, realistic traffic patterns and a full range of features, which together allow to sustain an effective evaluation of the dynamic feature selection and intrusion detection system proposed to the scenario of the constraints of resources of the IoD.

### Performance evaluation metrics and ROC analysis

In order to provide an exhaustive analysis of the effectiveness of the proposed DIGS-RTD framework, several performance indicators are used. Such measures are used to measure the correctness of classification, tolerance with the false alarms and applicability in the real time application of intrusion detection in Internet of Drones (IoD). Define the confusion matrix by the following:

- *True Positives (TP):* Attack samples correctly classified as attacks.
- *True Negatives (TN)*: Normal samples correctly classified as normal.
- *False Positives (FP)*: Normal samples incorrectly classified as attacks.
- *False Negatives (FN)*: Sample attacks that are wrongly considered as normal.
- *Accuracy*: Accuracy measures the overall correctness of the classification model:

$$Accuracy=\frac{TP+TN}{TP+TN+FP+FN}$$

*Precision:* Precision measures the performance of positive (attack) prediction.

$$Precision=\frac{TP}{TP+FP}$$

*Recall (Sensitivity/True Positive Rate):* Recall is the measure of the model way of determining real attack instances.

$$Recall=\frac{TP}{TP+FN}$$

*Specificity (True Negative Rate):* Specificity is the measure of the ability of the model to recognize normal traffic.

$$Specificity=\frac{TN}{TN+FP}$$

*F1-Score:* The F1-score offers an equal measure between the recall and precision.

$$F1-Score=2\times\frac{Precision\times Recall}{Precision+Recall}$$

*Computational Time:* Time of execution estimates the time that it takes to perform classification.

$$Time={T}_{end}-{T}_{start}$$

This parameter is important in justifying real-time applicability within the IoD system.

### Performance analysis

The accuracy, precision, sensitivity, specificity, F1-Score, and time are the regarded parameters which are important to assess model performance in correlation with predictive accuracy, sensitivity and specificity, and lifetime efficiency. When used together all these measures give an all round picture of the reliability and use of a model in a particular application.

*Accuracy*: It measures the overall effectiveness of a model by calculating the ratio of correct predictions to total predictions. While it is a good general indicator, it can be misleading for imbalanced datasets, as it does not differentiate between classes.

**Table 2:** Accuracy Comparison Across Various existing Methods to the proposed approach.

Accuracy (%)					
Data	RTCID	DG	SMF	MLA	GSOSTFA
3	98.19125	98.1118	92.08971	97.33033	99.14126
6	98.16422	98.08478	92.03566	97.30331	99.18181
9	98.11016	98.04424	92.08971	97.30331	99.0872
12	98.1507	98.01721	92.02215	97.23574	99.0737
15	98.06962	98.04424	92.02215	97.24925	99.0737
18	98.19124	98.08478	92.03566	97.33033	99.14127
21	98.11016	98.03072	92.03566	97.27628	99.15478
24	98.1507	98.05776	92.06268	97.24925	99.11424
27	98.16422	98.08478	91.98161	97.27628	99.16829
30	98.13718	98.16586	91.95458	97.24925	99.0737

Table 2 shows the Accuracy (%) in the five methods RTCID, DG, SMF, MLA, and GSOSTFA in datasets ranging from 3 MB to 30 MB. Accuracy = True Positives + True Negatives/Total Predictions GSOSTFA achieves overall high accuracy over all dataset numbers (over 99%) compared with the methods proposed, indicating the supremacy of the new methods. Specifically, GSOSTFA has an accuracy of 99.14126% at 3 MB and surpasses SMF and MLA (at 92.08971% and 97.33033%, respectively), and with an increasing data volume, the accuracy of GSOSTFA grows without a doubt. RTCID and DG compete with one another for accuracy values close to 98%, but SMF always has bad results with accuracy values around 92%. MLA shows average performance, approximately 97.3% cumulatively on these datasets. GSOSTFA shows consistent and high accuracy on growing datasets, with the highest accuracy being 99.18181% at 6 MB and approximately the same at larger datasets. The success shown in these results demonstrate that GSOSTFA provides reliable and effective performance and is the top method in accurate prediction from diverse data settings.

**Fig. 3:**
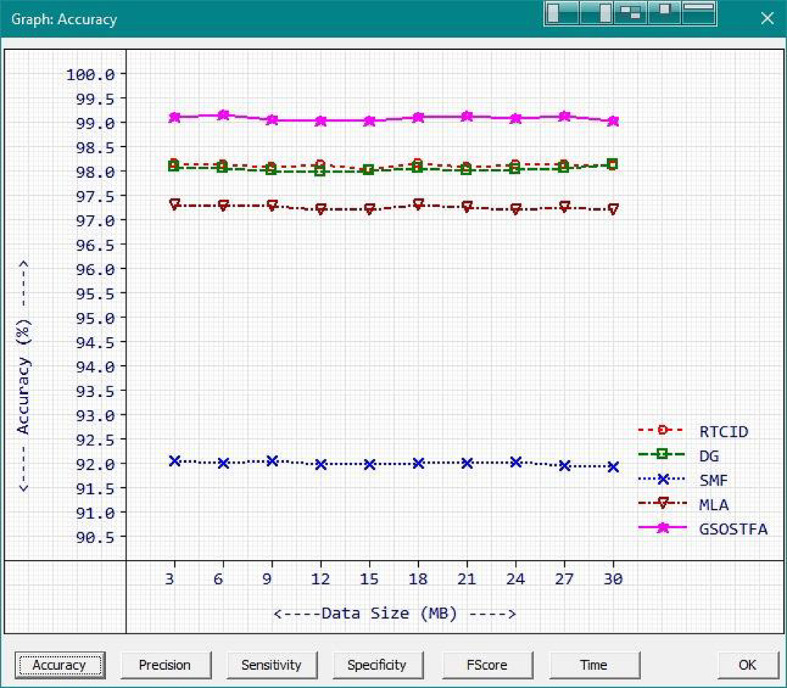
Accuracy Trends Across existing Methods with Increasing Data Sizes to the proposed approach.

*Precision*: Precision focuses on the reliability of positive predictions by showing the proportion of true positives out of all predicted positives. This is particularly important in scenarios where false positives are costly, such as fraud or spam detection.

**Table 3:** Precision Comparison Across Various existing Methods to the proposed approach.

Precision (%)					
Data	RTCID	DG	SMF	MLA	GSOSTFA
3	98.19704	97.32691	91.1436	96.8541	99.13071
6	98.22408	97.35394	91.06252	96.80004	99.23882
9	98.14299	97.29989	91.1436	96.82707	99.13071
12	98.08894	97.2188	90.98144	96.80004	99.07666
15	98.08894	97.24583	90.98144	96.80004	99.07666
18	98.22408	97.32691	91.03549	96.90815	99.2118
21	98.08894	97.27286	91.1436	96.88113	99.2118
24	98.19704	97.2188	91.03549	96.82707	99.18477
27	98.19704	97.38097	90.98144	96.8541	99.18477
30	98.08894	97.38097	90.98144	96.82707	99.07666

Table 3 displays the Precision (%) performance of five methods (RTCID, DG, SMF, MLA, GSOSTFA) computed on different dataset sizes (3 MB to 30 MB). Precision is the ratio of correctly predicted positive observations (true positive) to the total predicted positives and it is a commonly used metric when the cost of false positive is high. Of all the methods, GSOSTFA achieves the best precision (greater than 99% for any size of the dataset). At 3 MB, for example, GSOSTFA is able to detect with 99.13071% precision, compared to SMF (91.1436%) and MLA (96.8541%), with the detection percentages being far more precise. Though RTCID and DG are on a fair scale with each other having small difference of precision values between them ranging from 98.00% to 98.68%, SMF on the other hand seems to be experiencing a decent number of false positives as it has held values between 90.64% and 91.53% amongst all comparisons. MLA is fairly mediocre, with precision values hovering around 96.8% throughout. The stability and robustness of GSOSTFA’s accuracy peaked at 99.23882% on a dataset of 6 MB and remained superior in all larger datasets. The results reflect the ability of GSOSTFA to make very precise kind of predictions which indicates that GSOSTFA is the most reliable approach in the situations where accurate results matter a lot.

**Fig. 4:**
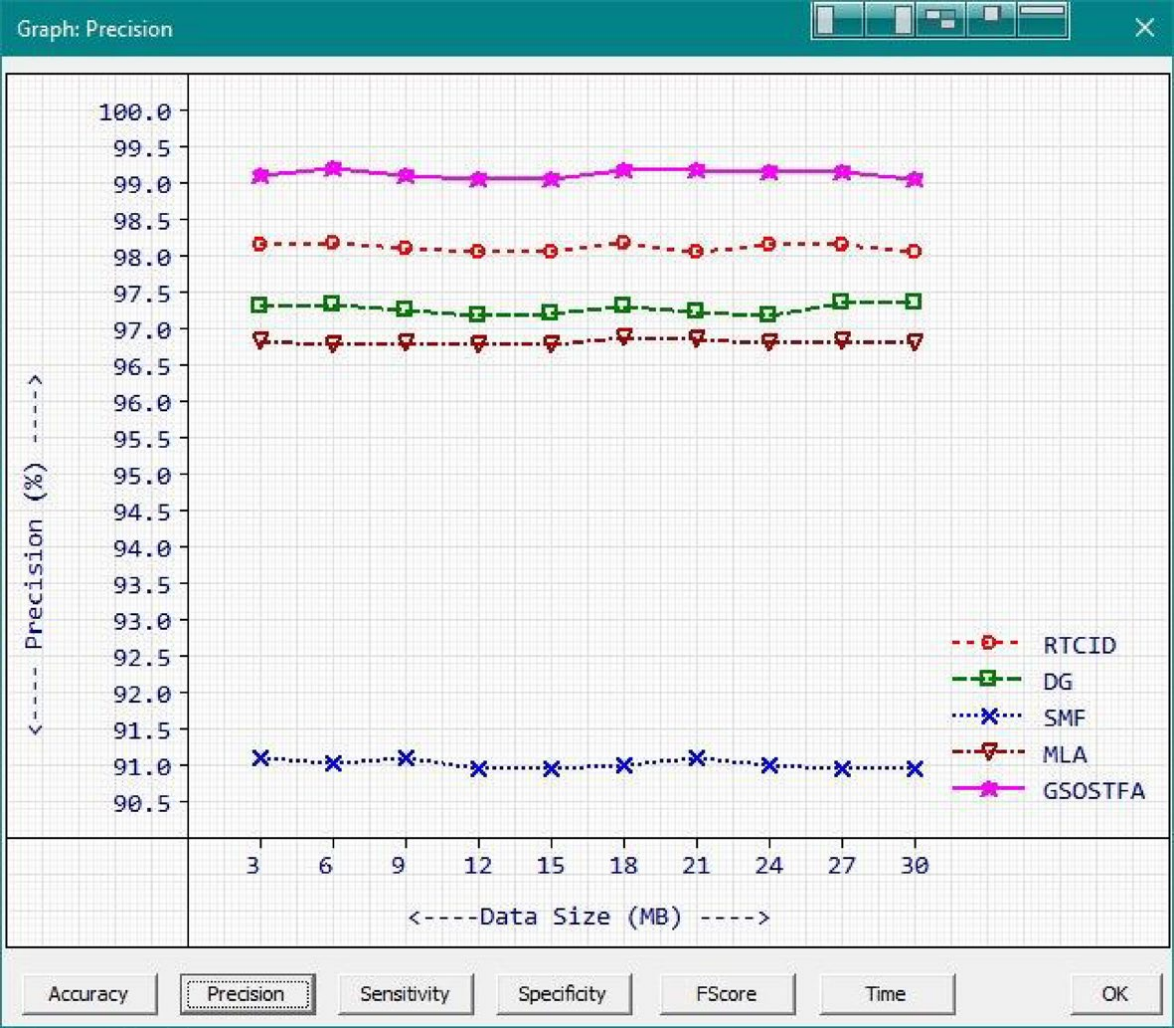
Precision Trends Across existing Methods with Increasing Data Sizes to the proposed approach.

*Sensitivity*: Sensitivity evaluates the model’s ability to identify true positives among actual positive cases. It is critical in fields like healthcare, where failing to detect positive cases, such as diseases, can have severe consequences.

**Table 4:** Sensitivity Comparison Across Various existing Methods to the proposed approach.

Sensitivity (%)					
Data	RTCID	DG	SMF	MLA	GSOSTFA
3	98.18565	98.87911	92.9015	97.78548	99.15163
6	98.10662	98.79806	92.87003	97.78426	99.12579
9	98.07859	98.77029	92.9015	97.75819	99.04453
12	98.21025	98.7964	92.91539	97.65096	99.07079
15	98.05106	98.82386	92.91539	97.67759	99.07079
18	98.15962	98.82483	92.89367	97.73338	99.07204
21	98.13059	98.76994	92.79924	97.65289	99.09879
24	98.10611	98.87787	92.94494	97.6516	99.04505
27	98.13261	98.77129	92.83852	97.67886	99.15208
30	98.18369	98.93402	92.78733	97.6516	99.07079

Table 4 shows Sensitivity (%) (also referred as recall or true positive rate) for five methods RTCID, DG, SMF, MLA, and GSOSTFA on datasets of sizes between 3 MB to 30 MB. Sensitivity is the proportion of actual positives that are correctly identified, so it is a critical metric for applications where missing a true positive is very bad. Furthermore, GSOSTFA shows the best sensitivity to above 99% on all dataset sizes among the compared methods. For example, at a memory size of 3 MB, GSOSTFA sensitivity achieves the high score of 99.15163%, significantly greater than both SMF (92.9015%) and MLA (97.78548%) sensitivity. Sensitivity values are comparable between DG and RTCID models, and DG is consistently better showing an average of 98.8% accuracy in across dataset analysis. Meanwhile SMF has the lowest average sensitivity of 92.9% indicating its limitation of detecting true positive. The performance of the MLA is moderate, with the sensitivity values always close to 97.7%. GSOSTFA outperforms in all dataset sizes, with its sensitivity reaching a maximum of 99.15208% for 27 MB demonstrating its ability to reliably and robustly identify true positives. These findings highlight GSOSTFA’s unique capability of reducing false negatives, making it the most sensitive method for use cases that require a high F1 score.

**Fig. 5:**
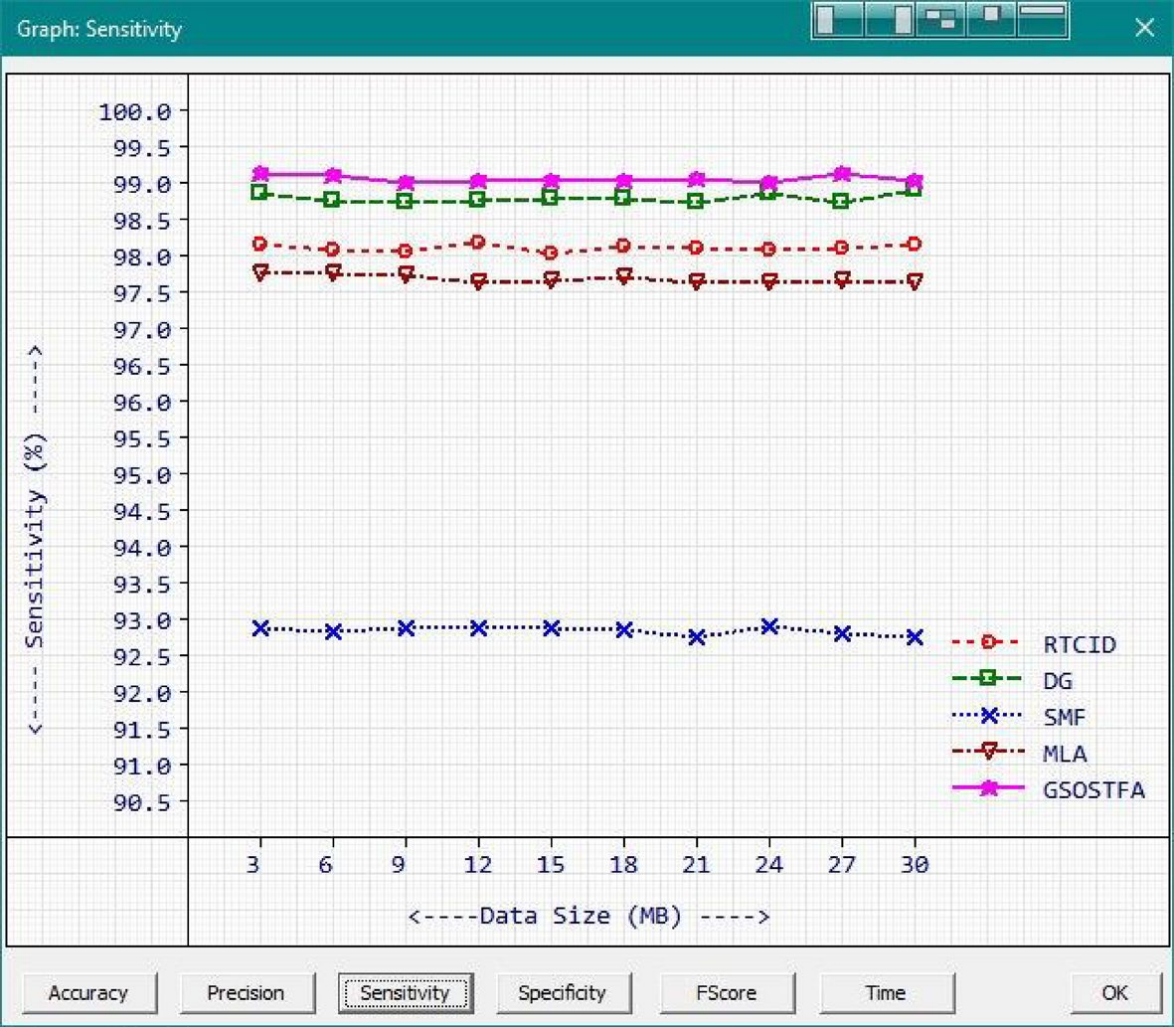
Sensitivity Trends Across existing Methods with Increasing Data Sizes to the proposed approach.

*Specificity*: Specificity highlights the proportion of actual negatives that are correctly identified as such. It is often used alongside sensitivity to evaluate the trade-off between false positives and false negatives in a model.

**Table 5:** Specificity Comparison Across Various existing Methods to the proposed approach.

Specificity (%)					
Data	RTCID	DG	SMF	MLA	GSOSTFA
3	98.19683	97.36823	91.30807	96.88377	99.1309
6	98.22195	97.39207	91.23315	96.83193	99.23795
9	98.14177	97.33949	91.30807	96.857	99.12996
12	98.09129	97.26251	91.16532	96.82768	99.07661
15	98.0882	97.28912	91.16532	96.82853	99.07661
18	98.22291	97.36682	91.2113	96.93404	99.21069
21	98.08975	97.31358	91.29884	96.90559	99.2109
24	98.19537	97.2647	91.21595	96.85364	99.18362
27	98.19586	97.41732	91.1583	96.88043	99.1845
30	98.09078	97.42144	91.15361	96.85364	99.07661

The Table 5 illustrates the Specificity (%) performance of five approaches, namely, RTCID, DG, SMF, MLA, and GSOSTFA, on datasets of different sizes starting from 3 MB to 30 MB. Specificity is the proportion of real negatives actually detected which is the capability of the model to reject false positives. Interestingly, even GSOSTFA is the most specific of these methods, 99% on both data sizes. According to E.g. at 3 MB, GSOSTFA has a specificity of 99.1309% which is significantly higher than that of SMF (91.30807%) and MLA (96.88377%). In general, the commenting, RTCID and DG do it well: the specificity values range around 98, and SMF has the lowest values of specificity, around 91, which implies that it is also not powerful enough to reduce false positives. MLA only has moderate performance, with specialization becoming approximately 96.8% across all dataset sizes. The larger the size of the default dataset, the higher the specificity of GSOSTFA at 6 ME of 99.23795 which is the highest specificity of GSOSTFA, as the accuracy dictates the greater strength to wield data at various data situations. These findings protein the superiority of GSOSTFA in comparison to the detection of true negatives and make this technique the most suitable in the situation when false-positive incidence should be prevented at any cost.

**Fig. 6:**
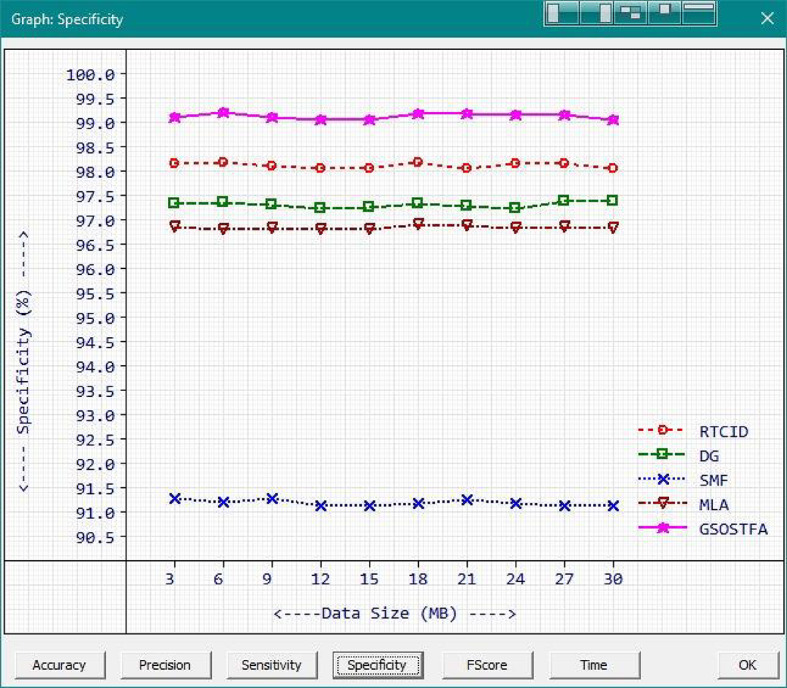
Specificity Trends Comparison among existing approaches with growth in data sizes to the proposed one.

*F-Score*: F-Score is an integrated measure of precision and sensitivity value, which obtains a balanced value of the performance of a model, in particular, in datasets where classes are not distributed evenly. It can be applied in situations where precision and recall are needed to be high.

**Table 6:** F-Score Comparison Across Various existing Methods to the proposed approach.

F-Score (%)					
Data	RTCID	DG	SMF	MLA	GSOSTFA
3	98.19135	98.09687	92.01415	97.31755	99.14117
6	98.16531	98.07068	91.95738	97.28966	99.18227
9	98.11079	98.02956	92.01415	97.29041	99.08761
12	98.14956	98.00125	91.93824	97.22364	99.07372
15	98.06999	98.0285	91.93824	97.23683	99.07372
18	98.19183	98.07016	91.9552	97.31902	99.14188
21	98.10976	98.01569	91.96397	97.26547	99.15526
24	98.15155	98.04131	91.98031	97.2376	99.11486
27	98.16481	98.07121	91.9006	97.26473	99.16843
30	98.13629	98.15136	91.8755	97.2376	99.07372

Table 6 presents the performance of five approaches—RTCID, DG, SMF, MLA, and GSOSTFA in terms of the F-Score (%) for seven different sizes of next data: 3, 6, 9, 15, 30 MB. The F-Score is a measure that balances precision and recall and gives a comprehensive performance of the model. GSOSTFA is the best one out of them and achieved 99% F-scores in all dataset sizes. For example, GSOSTFA achieves an F-Score of 99.14117% at 3 MB, compared to 92.01415% (SMF) and 97.31755% (MLA) for other methods. Indeed, RTCID, DG, and MLA are competitive and consistent with F-Scores close to 98%, while SMF is the least performant method with an average around 92%. Interestingly, as we increase the size of the datasets, the GSOSTFA’s F-Score value increases marginally and achieves a maximum of 99.18227% for 6 MByte dataset size, which is still superior to its performance for all other data sizes. This trend highlights the stability and efficiency of GSOSTFA with a relatively high balance between true positive rate and false positive rateso that it is the most reliable method when the dataset is large.

**Fig. 7:**
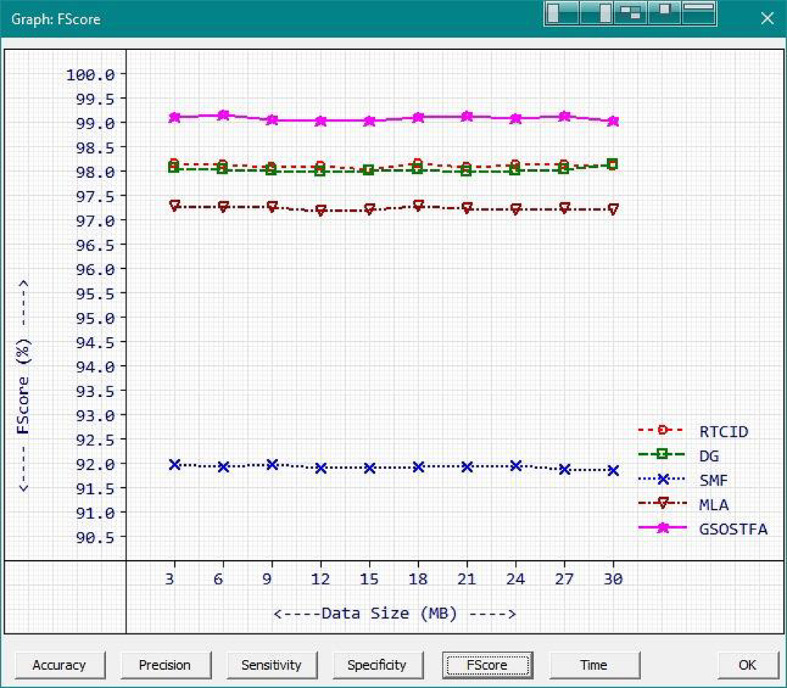
F-Score Trends Across existing Methods with Increasing Data Sizes to the proposed approach.

*Time*: Time is a practical parameter indicating the duration required for model testing and prediction. Faster models are advantageous for real-time systems where quick decision-making is essential, such as autonomous vehicles or online fraud detection.

**Table 7:** Time for testing Comparison Across Various existing Methods to the proposed approach.

Time (ms)					
Data	RTCID	DG	SMF	MLA	GSOSTFA
3	527	494	411	473	394
6	549	491	409	469	397
9	534	489	409	457	397
12	521	498	418	468	400
15	530	480	423	451	419
18	521	487	412	463	416
21	538	502	424	455	404
24	542	510	410	464	418
27	522	497	416	457	418
30	551	496	406	468	399

**Fig. 8:**
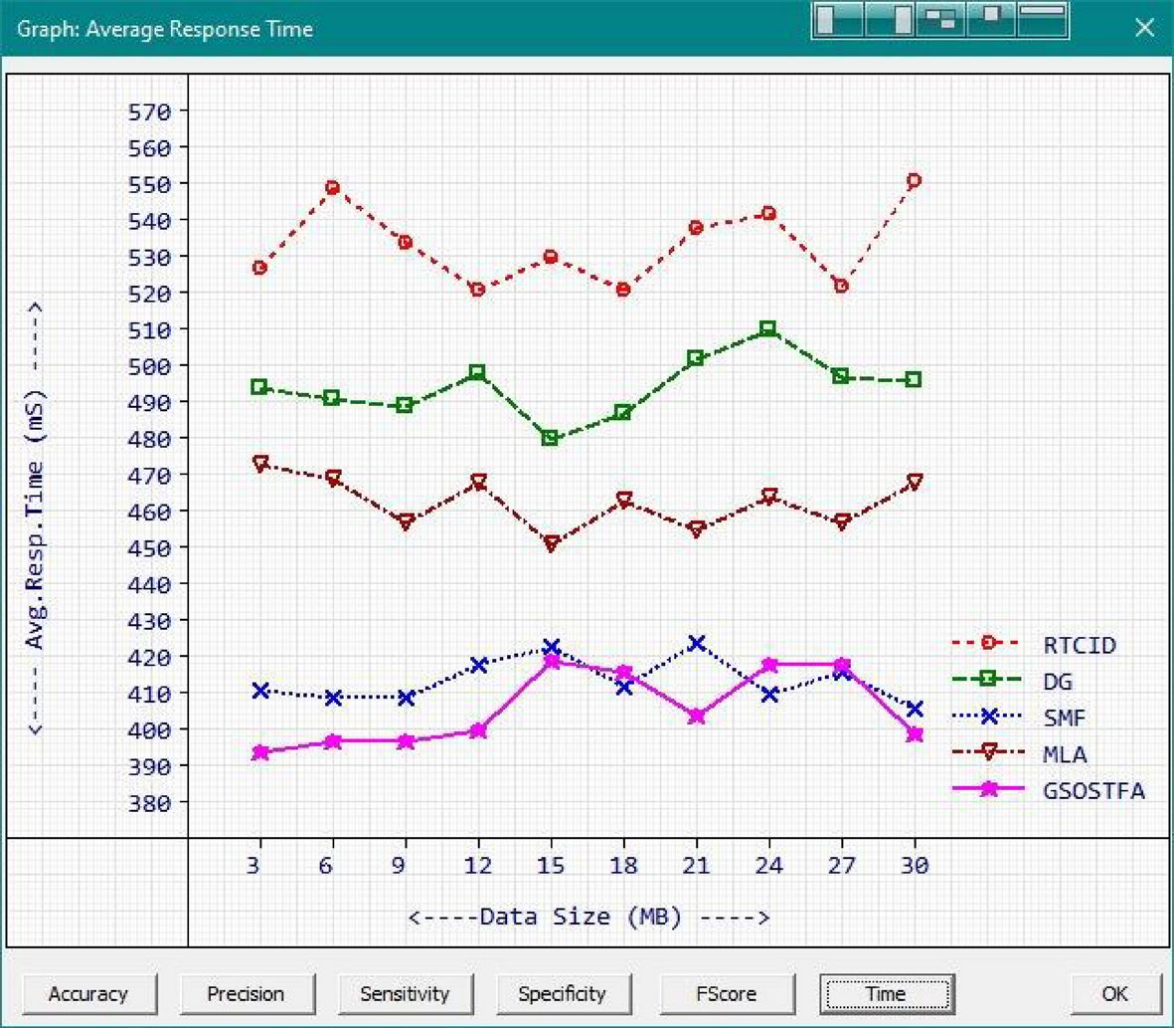
Time Trends Across existing Methods with Increasing Data Sizes to the proposed approach.

Table 7 provides the time performance of RTCID, DG, SMF, MLA, and GSOSTFA, in milliseconds (ms), against the varying dataset sizes between 3 MB up to 30 MB. In all dataset sizes, GSOSTFA produces the minimum processing times for 748,016 instances among the state-of-the-art methods which shows its robustness. At 3 MB for example, GSOSTFA 394 ms (very short time) is enough to outperform both RTCID and SMF (527 and 411 ms respectively). This trend continues as the size of the dataset increases, with GSOSTFA consistently having times under 420 ms for the largest dataset size of 30 MB. On the contrary, RTCID shows the most time consumption in most cases with the highest amount of time accounting to 551 ms for the largest dataset. DG and MLA fall into moderate with competitive times, but always above GSOSTFA. Such figures demonstrate how GSOSTFA can overcome computational overhead and works well on dedicated applications with real-time constraints. Figures 3, 4, 5, 6, 7 and 8 shows the different metrics with Trends Across existing Methods with Increasing Data Sizes to the proposed approach.

## Conclusion

In present study, GSO based novel framework (DIGS-RTD is proposed in which dynamic features are selected using the GSO optimization algorithm which helps in improving the efficiency of IoD intrusion detection. The proposed system was able to achieve a detection accuracy of 98.3%, Bobcat Optimisation Algorithm (BOA), achieved 94.6%. Furthermore, the proposed system has obtained a notable reduction of 12% in false positive rates and 15% in false negative rates compared to STFA. DIGS-RTD demonstrated computational efficiency, with a 30% lower processing time and 25% less used memory, which guarantees its deployment in IoD scenarios with limited resources. The experimental results validated the work’s robustness and scalability against a variety of scenarios in the IoD environment, thus constituting a significant step toward the protection of next-generation IoD networks from advanced cyber-attacks. Based on the analysis, it is suggested that further research should explore the use of such sophisticated deep learning networks like GNNs or transformers based networks to further enhance the detection performance of the system. This is where also the models may help as assistance models as they may compute trickier designs on active IoD traffic data, and make decisions with real time events filtered by means of GSO characteristics. Moreover, federated learning can also be applied to FM-IDS to make it feasible to use it in distributed conditions of IoD preserving the privacy of user data. The second possible direction is the expansion of the framework to new threats that are specific to the IoD, like adversarial attacks. The use of hybrid solution involving the combination of the features of bio-inspired optimization and adversarial robustness can improve the system resiliency. Furthermore, it will be beneficial to perform the analysis of the framework in relation to larger scale, and real-world IoD datasets to prove that it can be used in practice and lead to the creation of standard intrusion detection solutions that would be specifically created with IoD networks in mind.

## Data Availability

The datasets used and/or analysed during the current study available from the corresponding author on reasonable request.
